# Substituting Refined Flour with Soy Flour Improves Postprandial Glycemic Responses in Staple Foods Without Reducing Consumer Acceptability

**DOI:** 10.3390/nu18081173

**Published:** 2026-04-08

**Authors:** Stephanie I. Okoye, Rachel Carlson, Kenneth Dallmier, Marta Yanina Pepino

**Affiliations:** 1 Division of Nutritional Sciences, University of Illinois Urbana-Champaign, Urbana, IL 61801, USA; siokoye2@illinois.edu; 2 Northern Crops Institute, Fargo, ND 58105, USA; 3 Demand Side Ag, Mahomet, IL 61853, USA; ken.dallmier@demandsideag.com; 4 Food Science and Human Nutrition, University of Illinois Urbana-Champaign, Urbana, IL 61801, USA; 5 Carle Illinois College of Medicine, Urbana, IL 61801, USA

**Keywords:** soy flour replacement, glycemic response, insulin response, metabolic risk, consumer acceptance

## Abstract

**Background/Objectives**: Soy flour has been proposed as a functional ingredient to improve the protein and fiber content of foods; however, its metabolic and sensory effects, particularly in individuals at elevated risk for metabolic disease, remain insufficiently characterized. This randomized, repeated-measures study examined whether substituting refined wheat or corn flour with soy flour influences postprandial glucose and insulin plasma concentrations, appetite ratings, and product acceptability in adults with overweight or obesity. **Methods**: Participants (*N* = 17) attended at least three separate visits during which they consumed, in random order, a food matrix with 0% (control), 10%, or 30% soy flour substitution. Food matrices included breads (*n* = 10), tortillas (*n* = 10), and arepas (*n* = 8); some participants completed more than one matrix. Postprandial plasma glucose and insulin concentrations were measured at baseline and at 15, 30, 60, 90, and 120 min post-ingestion. Subjective hunger, satiety, and product liking were assessed using a 10 cm visual analog scale. **Results**: Compared with the control condition, substituting 30% of refined flour with soy flour significantly reduced the area under the concentration–time curve for postprandial glucose for breads (*p* = 0.03) and arepas (*p* = 0.04), and reduced plasma glucose concentrations at 90–120 min for tortillas (*p* = 0.0009). In contrast, postprandial insulin concentrations and subjective hunger and satiety ratings did not differ across substitution levels or food matrices (all *p* > 0.05). Importantly, even 30% soy flour substitution maintained product liking. **Conclusions**: Incorporating up to 30% soy flour may improve postprandial glycemic responses without compromising overall liking, supporting its potential as a practical food reformulation strategy to improve metabolic health in populations at increased risk of metabolic disease.

## 1. Introduction

Obesity remains a major global health challenge. In the United States, its prevalence has risen steadily since the 1970s, affecting nearly 40% of adults by 2016 and projected to approach 50% by 2030 [1,2]. The condition contributes to numerous chronic diseases, including type 2 diabetes, cardiovascular disease, certain cancers, and reduced quality of life [3,4]. Although obesity may arise from multiple risk factors (genetic, behavioral, environmental, and psychosocial factors), diet remains among the most modifiable determinants [5]. Consequently, strategies that improve dietary quality have become a major focus in efforts to prevent or reduce the burden of metabolic disease. In particular, the widespread consumption of energy-dense foods rich in refined carbohydrates has been identified as a key dietary contributor to the epidemic, highlighting the need for effective strategies to reduce their intake [6].

Refined carbohydrates are commonly consumed in staple foods made from refined flours, such as white breads and tortillas. These foods are typically rapidly digested, elicit sharp postprandial increases in blood glucose and insulin, and provide limited satiety [7,8]. In addition to their high glycemic properties, they are often poor sources of essential nutrients, including protein, which makes them lower-quality carbohydrate options. In contrast, foods that elicit lower glycemic responses, such as legumes, are considered higher-quality carbohydrate sources [7,8]. Soybeans, a widely consumed legume, are rich in plant-based protein, dietary fiber, vitamins, minerals, and diverse bioactive phytochemicals [9]. Given these nutritional benefits, partial replacement of refined wheat flour with soy flour appears to be an attractive strategy to improve nutrient composition and attenuate postprandial glycemic responses in commonly consumed foods. However, despite these advantages, some negative sensory characteristics of soy-enriched products must also be considered. Achieving a balance between metabolic benefits and consumer acceptability is therefore crucial for successful formulation.

Previous studies suggest that low-to-moderate levels of soy flour substitution (up to 15%) in refined wheat flour-based products can enhance nutritional value while maintaining desirable sensory characteristics [10,11,12]. Similarly, substituting up to 15% of breads formulated with corn flour with soy flour preserves consumer acceptance [13]. From a metabolic perspective, Mirzaei et al. demonstrated that, in healthy women, substituting 25% or 50% of refined wheat flour with soy flour lowered glycemic load and more than doubled satiety compared with control bread [14]. Other studies replicated the reduced glycemic responses after consumption of breads with increasing soy flour substitution within 10 to 20% [15] or other food matrices, such as pretzels [16]. Together, these findings indicate that higher substitution levels of refined wheat or corn flour with soy flour may yield greater metabolic benefits but may also compromise sensory qualities and acceptability of these food products when replacement is above 15%. Of importance, the previous studies mentioned above excluded people with obesity, and it is unclear if the same metabolic benefits are observed in these individuals, who typically have more insulin resistance. Thus, the substitution level that produces meaningful improvements in postprandial glucose excursions in people at higher metabolic risk, and that maintains consumer acceptability, remains insufficiently characterized.

The present study addresses this uncertainty by evaluating the effect of soy flour substitution at 10% and 30% levels in culturally relevant staples, breads, tortillas, and arepas (a corn-based food). We assessed the effects of this substitution on postprandial glucose, insulin, satiety, and hedonic liking in adults with overweight or obesity. We hypothesized that relative to control formulations, increasing soy flour substitution would attenuate postprandial glucose responses without impacting insulin responses. As a secondary hypothesis, we postulate that higher substitution would enhance satiety and reduce hunger but may lower product liking due to sensory trade-offs.

## 2. Materials and Methods

### 2.1. Participants

**Figure 1:**
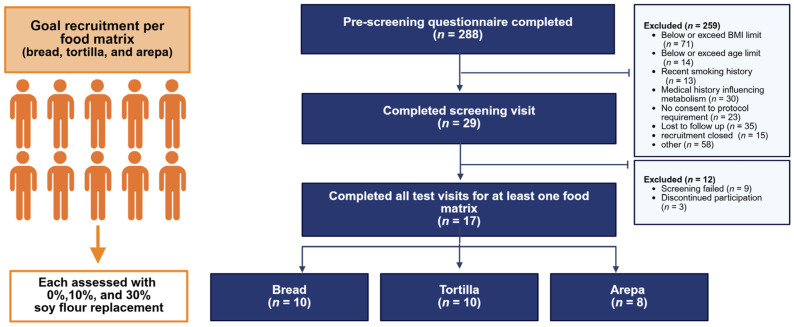
Flow diagram of study participants. Some participants completed test for multiple food matrices. Specifically, the tortilla food matrix tests included 5 participants who also completed the bread tests, and the arepa food matrix tests included 3 participants who also completed the bread and tortilla tests, and 3 participants who completed the tortilla test.

Between February 2024 and June 2025, 17 adults were recruited and they completed all three test visits for at least one and up to three of the food matrices. For this randomized, crossover, controlled trial, we aim to assess 10 participants for each of the food matrices (i.e., 10 for breads, 10 for tortillas, and 10 for arepas, Figure 1); however, due to budgetary constraints, we capped recruitment at 8 for arepa (which was the last food matrix we studied).

We included both male and female between 21 and 45 years of age with a body fat% > 30% for females or >20% for males and a body mass index (BMI) ≥ 25 kg/m^2^ and <40 kg/m^2^ (i.e., overweight and obesity, excluding severe obesity). All races/ethnicities were welcome to participate. Exclusion criteria were being pregnant, breastfeeding or post-menopausal, having gluten intolerance (including gluten allergy, wheat allergy, celiac disease), soy intolerance or soy allergy, a history of bariatric surgery, diabetes (fasting glucose level ≥ 126 mg/dL or plasma glucose level 2 h after an oral glucose tolerance test > 199 mg/dL), or taking medicines to treat diabetes, polycystic ovary syndrome, untreated hypertension, anemia, malabsorption syndrome, inflammatory intestinal disease, liver, or kidney disease, or taking any medication that might affect glucose metabolism or the results of our study. Additional exclusion criteria were smoking nicotine or having quit smoking nicotine (cigarettes, vapes, or E-cigarettes) for less than 6 months, a history of seizures or cancer < 5 years ago, or abnormalities in the metabolic panel test assessed during screening (e.g., liver enzymes > 2 times the upper limit).

All study procedures were approved by the Institutional Review Board at the University of Illinois Urbana-Champaign (UIUC protocol number: # 24388 (Original format approved 18 August 2023) and #24-0303 (new electronic record)) and registered on ClinicalTrials.gov (NCT06280625) on 20 February 2024. All participants signed an informed consent form before any study procedure took place. Figure 1 shows a flow diagram of participants contacted for this study and their final inclusion/exclusion status in the data analysis.

### 2.2. Procedure

Before recruitment, participants first attended a screening visit to determine eligibility. All eligible individuals were then invited to complete three additional metabolic visits, each corresponding to one of the soy flour replacement levels (0%, 10%, or 30%). The order of the 0%, 10%, and 30% was randomized across visits using an online randomization tool and participants were blinded to the sequence. Testing began with the breads; once assessment for that matrix was completed, testing proceeded with the tortillas and arepas, respectively. Due to budgetary constraints encountered during the final phase of the study, the arepa matrix was completed in 8 rather than the planned 10 participants. In addition, plasma insulin concentrations were not measured for the 10% arepa condition, and only the available arepa conditions were included in the final analyses. After completing testing for one food matrix (i.e., all three soy flour levels), participants had the option to continue in the study and complete testing for additional food matrices, at least one month after their previous set of visits.

Prior to each session, participants were instructed to refrain from eating or drinking anything other than water for 12 h overnight. They were also asked to maintain their usual diet and avoid strenuous exercise for at least three days before each study visit, and to abstain from caffeine and alcohol for at least two days. At the start of the first visit, anthropometric measurements (height and weight) were recorded to calculate BMI, and weight was reassessed at subsequent visits. Participant vital signs and plasma glucose were monitored at each visit by trained personnel. Female participants completed a urine pregnancy test at the start of each session to confirm non-pregnant status. Each metabolic visit lasted approximately 3–4 h, and participants received monetary compensation for their participation.

### 2.3. Screening Visit

Participants arrive at the laboratory at UIUC after 12 h of overnight fasting at home. In addition to BMI determination, we performed a body composition test using the BOD POD machine to measure body fat percentage. To exclude for diabetes and other metabolic conditions, blood samples were drawn at baseline and at several times during a 2 h oral glucose tolerance test (OGTT), along with a metabolic panel and a complete blood count test.

**Figure 2:**
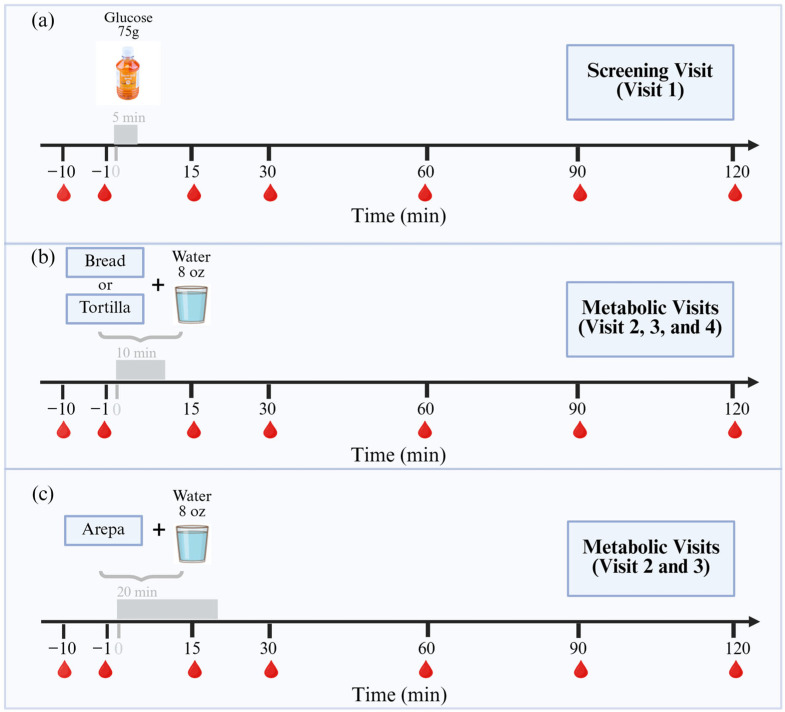
Study procedures for screening and metabolic visits. The image illustrates the timeline of events for (**a**) Oral Glucose Tolerance Test in the screening visit; (**b**) Bread and Tortilla test visits, and (**c**) Arepa test visits.

For the blood test and OGTT, an intravenous catheter was placed in a hand vein while the participant was in a recumbent position. After catheter placement, the hand was heated in a warming box to obtain arterialized venous blood samples. In few cases where we were unable to place the catheter in a hand vein, we placed it in a vein in the arm (but we used the same catheter location for all metabolic study visits). We collected seven blood samples in chilled EDTA tubes containing a protease inhibitor cocktail. Two samples were drawn before (time—10 min and time—1 min), and the remaining at 15, 30, 60, 90, and 120 min after drinking 75 g of glucose (Figure 2a). After the last blood draw of the OGTT, participants were given a sample of the control bread, tortilla, or arepa (all in their original recipes with 0% soy flour) to assess the acceptability of these control products.

### 2.4. Metabolic Study Visits

The three separate metabolic study visits following the screening visit were scheduled approximately 7 days apart. Like for the screening visit, participants arrived after a 12 h overnight fast at home. For the intravenous catheter placement and the blood draws, we followed the same procedures as those described above for the screening visit. At time zero, participants were asked to consume the food products and an 8-oz cup of water within 10 min (Figure 2b). However, in the case of arepa, after pilot testing and experience with the first study participant, we had to extend the period of food consumption to 20 min (Figure 2c).

### 2.5. Food Test Products

Illinois soybeans were sourced from Clarkson Grain Co., Inc. of Cerro Gordo, IL, USA. A clear hilum soybean was selected to remove any possibility of discoloration of the test product due to dark hilum. The soybeans were cleaned and color-sorted to remove any foreign matter. Defatted soybean flour was manufactured by the Northern Crop Institute (NCI). The whole soybeans were dehulled and flaked, then hexane-extracted to produce defatted soybean meal. The meal was then dried and ground to produce food-grade soybean flour. A target Protein Dispersibility Index of 90% ensured active enzymes and good dough for the products.

Recipes for white wheat breads, wheat tortillas, and corn arepas were developed and validated by NCI. Depending on the food matrix, soy flour was substituted for wheat flour (breads and tortillas) or corn flour (arepas) on a weight-to-weight basis. All recipes maintained approximately the same calorie content across the three levels of replacement (i.e., 0%, 10%, 30%) within each food matrix, and the portion for each food matrix at 0% level of replacement included approximately 70 g of carbohydrates (Table 1). Finished products were sent frozen in individual portions to the laboratory at the UIUC. Upon receipt, all food products were stored in a food-grade freezer, and portions to be used for each study visit were thawed the night before each visit.

### 2.6. Glucose and Insulin Responses

Plasma glucose concentration for screening and metabolic visits was measured at the bedside using a biochemistry analyzer (YSI International, Yellow Springs, OH, USA), and plasma insulin concentration was measured using an Elecsys Insulin assay (Rosche Diagnostics, Indianapolis, IN, USA) at the Core Laboratory at Washington University in St. Louis.

### 2.7. Visual Analog Scales

Participants were asked to rate their liking of the food product consumed during each metabolic study visit using a 10 cm visual analog scale (VAS) anchored at “not at all” (0) and “a lot” (10). They also completed 10 cm VASs to assess their perceived levels of hunger and satiety, responding to the questions “How hungry do you feel?” and “How satiated are you?” [17]. The hunger scale ranged from “not hungry at all” (0) to “never been more hungry” (10), while the satiety scale ranged from “totally empty” (0) to “cannot eat another bite” (10). Ratings for hunger and satiety were recorded at baseline and at every subsequent time point when a blood sample was drawn during each metabolic visit.

### 2.8. Data Analysis

Areas under the plasma concentration–time curves (AUC) for glucose and insulin were calculated using the trapezoidal method [18]. The effects of replacing regular flour or corn flour with soy flour at 10% and 30% on plasma glucose and insulin concentrations, glucose and insulin AUCs, time to peak, and peak glucose and insulin responses, as well as on hunger and satiety ratings, were evaluated using separate repeated-measures analyses of variance (ANOVAs) for each outcome variable and each food matrix (bread, tortilla and arepa). For each analysis, the within-subject factors were replacement level (0%, 10%, and 30%; except for arepa, for which only 0% and 30% were tested) and when applicable, time (0, 15, 30, 60, 90, and 120 min). Liking scores were analyzed using one-way ANOVAs for each food matrix. Because liking data collection began after the study had started, complete liking scores were available for only 6 of the 10 participants for the breads and for 7 of the 8 participants for the arepas due to one technical error; however, all 10 participants provided liking scores for tortillas. When statistically significant differences were detected Fisher’s least significant difference tests were used for post hoc comparisons.

Sample size calculations were conducted in G*Power version 3.1 for the planned repeated-measures design. We specified a repeated-measures ANOVA with two within-subject factors: replacement level (0%, 10%, 30%) and time (0, 15, 30, 60, 90, and 120 min). This within-subject approach improves statistical efficiency by controlling for inter-individual variability in glucose metabolism. Based on prior studies in healthy-weight adults showing lower postprandial glucose after consumption of products containing > 10% soy replacement compared with 0% soy flour controls [15], we assumed a moderate effect size (f = 0.25). With α = 0.05 and 80% power, the estimated minimum sample size to detect differences in relative glucose concentrations across conditions was 10 participants for each food matrix.

## 3. Results

### 3.1. Subject Characteristics

We completed all three levels of replacement (0%, 10%, and 30%) in 10 participants for breads and tortillas, but only 8 participants completed arepas, and only plasma samples from control (0%) and 30% replacement were analyzed (Figure 1; Table 2). Participants’ characteristics are shown in Table 2.

### 3.2. Plasma Glucose

**Figure 3:**
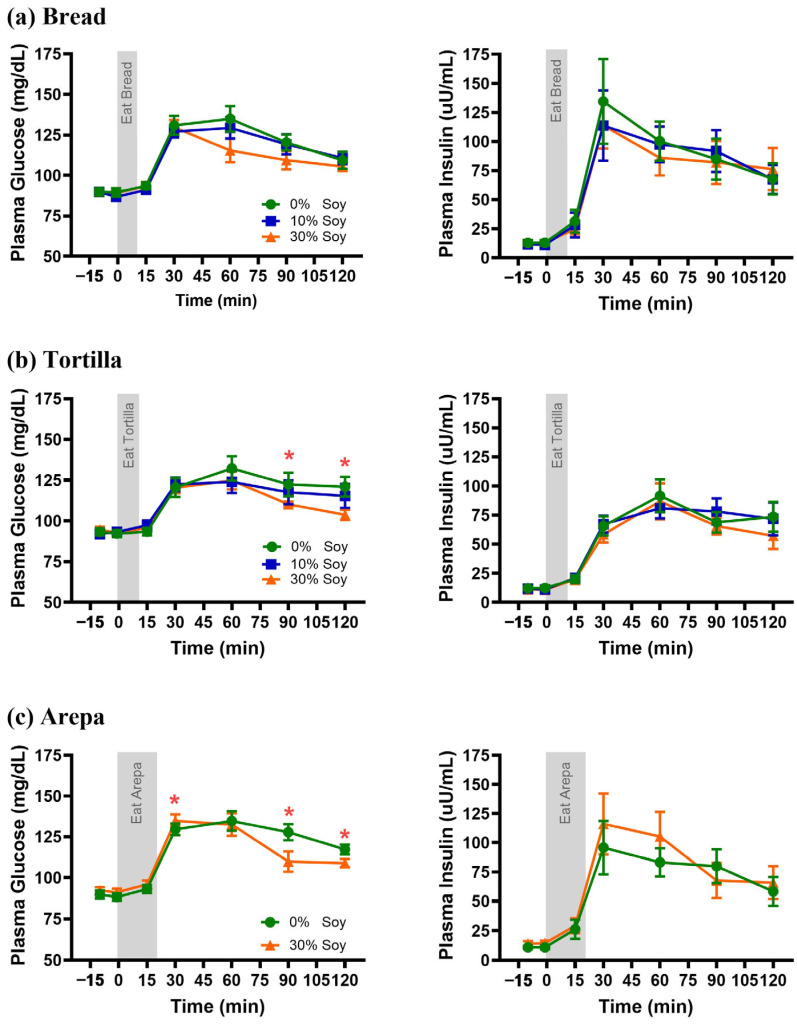
Postprandial plasma glucose (**left panels**) and plasma insulin (**right panels**) concentrations following consumption of products with varying levels of soy flour. Responses are shown for (**a**) breads, (**b**) tortillas, and (**c**) arepas prepared with 0% soy flour (green circles), 10% soy flour (blue squares), or 30% soy flour (orange triangles) substitution. Data are presented as mean ± SEM. Shaded areas indicate the eating period. * Significant difference between 30% soy flour and control (0%) at individual time points (*p* < 0.05).

Plasma glucose concentrations were generally lower following consumption of food staples containing 30% soy flour compared with the 0% control (Figure 3). For the breads, there was a main effect of replacement level such that consumption of the 30% soy flour bread resulted in overall lower plasma glucose concentrations (soy%: *F_(_*_2,18)_ = 17.4, *p* = 0.05; soy% × time: *F_(_*_12,108)_ = 2.04, *p* = 0.03), a reduce glucose peak (*F_(_*_2,18)_ = 5.6, *p* = 0.01), and a smaller plasma glucose AUC than the 0% control (*F_(_*_4,18)_ = 4.4, *p* = 0.03). The 10% soy bread produced intermediate plasma glucose concentrations that were not significantly different from either 0% or 30%. For tortillas and arepas, significant replacement level × time interactions were observed (soy% × time: tortilla *F_(_*_12,108)_ = 3.08, *p* = 0.04; Arepa *F_(_*_6,42)_ = 10.3, *p* = 0.0007) indicating that plasma glucose concentrations were lower between 90 and 120 min after consumption of the 30% soy formulation compared with control (Figure 3b,c). Plasma glucose AUC was also reduced following the 30% soy arepa compared with control (*F_(_*_1,7)_ = 6.1, *p* = 0.04), whereas no significant difference was observed between the 30% soy tortilla and control tortilla (*F*_(2,18)_ = 2.16, *p* = 0.1) (Table 3).

### 3.3. Plasma Insulin

Across all food matrices, plasma insulin concentrations increased postprandially (main effect of time, all *p* < 0.001). However, replacement of wheat or corn flour with soy flour did not affect postprandial plasma insulin concentrations (all *p* > 0.3; Figure 3, right panels) or insulin AUCs for any food matrix (Table 4). For arepas, inclusion of 30% soy flour resulted in a shorter time to peak insulin compared with the 0% control formulation, without a significant difference in peak insulin concentration (Table 4).

### 3.4. Liking Ratings for the Different Products

**Figure 4:**
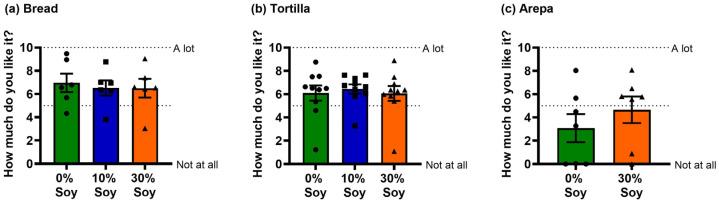
Liking ratings following consumption of products formulated with varying levels of soy flour. Visual analog scale (VAS) liking scores for (**a**) breads, (**b**) tortillas, and (**c**) arepas prepared with 0%, 10%, or 30% soy flour substitution. Bars represent mean ± SEM; symbols denote individual participant ratings. Higher values indicate greater liking.

Soy flour substitution did not affect liking ratings for breads or tortillas (bread *F_(_*_2,10)_ = 0.8, *p* = 0.5; tortilla *F_(_*_2,18)_ = 0.4, *p* = 0.7; Figure 4). For arepas, there was a trend toward higher liking ratings for the 30% soy flour formulation compared with the control (*F_(_*_1,6)_ = 4.6, *p* = 0.08; Figure 4).

### 3.5. Hunger and Satiety Ratings

**Figure 5:**
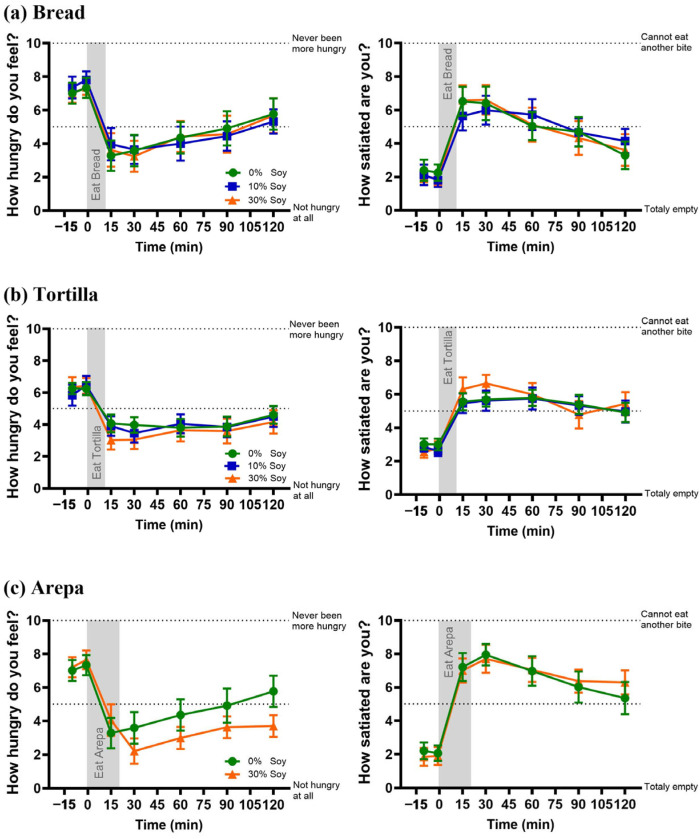
Hunger and satiety ratings following consumption of products formulated with varying levels of soy flour. Hunger (**left panels**) and satiety (**right panels**) visual analog scale (VAS) scores over time for (**a**) breads, (**b**) tortillas, and (**c**) arepas prepared with 0%, 10% or 30% soy flour substitution. Data are presented as mean ± SEM. Shaded areas indicate the eating period. Higher hunger scores indicate greater hunger, and higher satiety scores indicate greater fullness.

Consumption of all food matrices decreased hunger and increased satiety ratings (main effect of time, all *p* < 0.001). However, replacement of wheat or corn flour with soy flour did not affect either outcome (all *p* > 0.05; Figure 5).

## 4. Discussion

This randomized, repeated-measures study demonstrates that partial substitution of refined wheat or corn flour with soy flour improves postprandial glycemic responses in commonly consumed staple foods without compromising acceptability in adults with overweight or obesity. Replacing 30% of refined flour with soy flour attenuated postprandial glucose responses across breads, tortillas, and arepas, extending previous evidence that reformulation strategies targeting macronutrient composition can modulate glycemic exposure in mixed meals. The reduction in postprandial glucose was not accompanied by higher insulin concentrations, suggesting improved glycemic control without increased insulin demand, and supporting the possibility that soy flour substitution influenced not only the nutrient profile of the foods but also the rate of postprandial glucose appearance. Soy flour substitution did not significantly affect hunger, satiety or liking ratings, for any product.

The lower postprandial glucose excursions observed in the present study are consistent with prior reports in individuals with normal weight, showing that soy flour-enriched foods reduce glycemic responses compared with refined wheat flour controls [14,15,16]. The current findings extend this evidence to adults with overweight or obesity, a population at elevated risk for metabolic disease. Prior studies by Haque et al., Mirzaei et al., and Simmons et al. reported improvements in glycemic index primarily at soy flour replacement levels exceeding 10%. In line with these findings, the present study showed a greater reduction in plasma glucose AUC with 30% soy replacement, whereas 10% substitution produced smaller, nonsignificant effects. Notably, the magnitude and timing of glycemic responses differed across food matrices, suggesting that the metabolic impact of soy flour incorporation may depend not only on macronutrient composition but also on structural characteristics of the food matrix that influence digestion and glucose appearance after the meal. 

Growing evidence emphasizes the importance of evaluating both glycemic and insulin responses when assessing the metabolic effects of food reformulation strategies, particularly in populations at elevated metabolic risk [19]. In the present study, soy flour substitution did not alter postprandial insulin responses, consistent with findings by Simmons et al., who reported comparable insulin responses following consumption of wheat flour pretzels and pretzels containing 27% soy flour [16]. The absence of differences in insulin concentrations despite lower glucose exposure suggests improved glycemic handling without increased insulin demand. Although this pattern is partly consistent with lower available carbohydrate content of the soy-substituted foods, other physiological mechanisms may also have contributed. Higher protein content, as that provided by soy flour [16,20], may slow gastric emptying and reduce the rate at which glucose appears in the circulation after the meal [21]. In addition, the incorporation of soy flour may modify the food matrix through starch–protein interactions, thereby affecting starch gelatinization, microstructure, and enzyme accessibility, which could influence the rate of carbohydrate digestion and absorption [22]. These mechanisms may help explain why glycemic attenuation was observed without a parallel rise in circulating insulin. However, because gastric emptying, intestinal glucose absorption kinetics, and incretin responses were not measured in the present study, these explanations should be considered plausible interpretations and should be investigated in future studies.

Evidence from animal models also supports a glucose-lowering effect of soy-based dietary interventions. In rats, soy protein has been shown to improve glucose tolerance and insulin sensitivity relative to casein, and to enhance insulin-stimulated glucose utilization in insulin-resistant models [23,24]. However, these studies differ in many important ways from the present trial because they generally used chronic feeding designs, purified soy protein, or experimental rodent models rather than acute substitution of soy flour into commonly consumed staple foods. Accordingly, those pre-clinical findings provide mechanistic support for the potential metabolic benefits of soy, but they are not directly comparable to the present postprandial crossover data in adults with overweight or obesity.

In contrast with some previous reports showing enhanced satiety following soy flour substitution [14], the present study did not detect significant changes in satiety ratings with soy flour replacement of up to 30% in any of the foods tested. Differences in study populations may partially explain this discrepancy, as our participants were adults with overweight or obesity, a group characterized by altered appetite regulation and metabolic responses. One possible explanation relates to the interplay between glycemic and insulinemic responses in appetite control. Caferoglu et al. reported that foods characterized by both low glycemic and low insulin responses elicited greater satiety in individuals with insulin resistance compared with foods producing low glycemic but higher insulin responses [19]. Because insulin concentrations did not differ across soy substitution levels in the present study, the absence of satiety effects may reflect limited changes in the hormonal signals associated with appetite regulation. Further research is needed to clarify how glycemic and insulinemic characteristics of reformulated foods influence subjective satiety.

Several limitations should be considered when interpreting these findings. Although prior studies have often reported acceptable sensory outcomes with soy flour replacement approximately 10% [10,11,25] the present study observed maintained liking even at 30% substitution across food matrices. However, because only 10% and 30% replacement levels were evaluated, the present design did not permit fine-grained characterization of the dose–response relationship or identification of the lowest effective substitution level required to achieve metabolic benefits, and future studies aimed at refining the lowest effective substitution level would be valuable. Sample size for the repeated-measures metabolic analyses was determined a priori, and the planned enrollment target (*n* = 10 per matrix) was achieved for breads and tortillas, supporting adequate power for those primary comparisons. However, because the arepa matrix was studied last, budget limitations restricted enrollment to eight participants, resulting in a less complete dataset for that matrix. This limitation may reduce the precision and generalizability of the arepa findings. Insulin concentrations were not measured for the 10% arepa condition due to funding constraints restricting evaluation of potential dose–response effects for this product. Additionally, glycemic index values were not calculated, limiting direct comparison with studies reporting glycemic index outcomes. However, the primary aim of this study was to directly assess the postprandial metabolic response to soy flour substitution through plasma glucose and insulin concentrations. We considered this approach particularly relevant in adults with overweight or obesity, because glycemic index reflects only the glucose response under standardized conditions and does not capture the accompanying insulin response. Food liking ratings were introduced later in the protocol, resulting in incomplete sensory data for some participants. Expanded sensory evaluation using trained panels alongside consumer testing would further clarify how soy flour influences flavor, and specific sensory attributes, including texture, aroma, and overall acceptability across different food matrices. Findings from those studies could help refine formulations to optimize both metabolic benefits and consumer acceptance. Because the present study assessed only acute responses to a single meal, longer-term intervention studies are also warranted to determine whether habitual consumption of soy-enriched staple foods leads to sustained improvements in glycemic control, insulin sensitivity, appetite regulation, and overall diet quality in populations at elevated metabolic risk.

## 5. Conclusions

Partial substitution of refined wheat or corn flour with soy flour at a 30% level reduced postprandial glycemic responses across multiple staple foods in adults who are overweight or obese, supporting the metabolic potential of macronutrient-focused food reformulation strategies. These findings extend prior work conducted in healthy populations by demonstrating comparable glycemic benefits in individuals at elevated metabolic risk and across diverse food matrices. Together, the results highlight soy flour incorporation as a feasible approach to improving postprandial glycemic control while maintaining overall liking.

## Figures and Tables

**Table 1:** Food composition.

Food Matrix	Bread			Tortilla			Arepa	
Soy %	0%	10%	30%	0%	10%	30%	0%	30%
Net sample weight (g)	150.0	150.0	150.0	160.0	160.0	166.0	280.0	305.0
Net sample calories (kcal)	372.0	372.0	372.0	443.2	443.2	453.0	338.8	357.4
Macronutrients (g)								
Carbohydrates	70.5	66.0	58.5	68.8	65.6	58.0	70.0	61.1
Protein	9.0	13.5	21.0	9.6	12.8	21.6	8.4	21.4
Fat	6.0	6.0	6.0	14.4	14.4	14.9	2.8	3.1

**Table 2:** Participants’ characteristics.

Food Matrix	Bread	Tortilla	Arepa
Age, years (SD)	31.8 (4.4)	32.0 (6.3)	34.1 (5.4)
Sex, *n*			
Male	5	5	5
Female	5	5	3
Race, *n*			
White	7	6	7
Asian	3	4	1
Weight, kg (SD)	93.7 (16.7)	89.2 (11.7)	93.3 (11.6)
Height, m (SD)	1.75 (0.12)	1.71 (0.09)	1.75 (0.08)
Body mass index (BMI), kg/m^2^ (SD)	30.5 (3.2)	30.3 (2.3)	30.5 (2.5)
Fat Percent, % (SD)			
Male,	28.7 (3.4)	30.7 (8.8)	34.0 (7.6)
Female,	43.4 (5.0)	38.5 (4.1)	36.9 (4.6)
Average Fasting Glucose, mg/dL (SD)	87.8 (6.4)	93.3 (7.0)	90.1 (4.7)
Average 2 h Glucose, mg/dL (SD)	140.8 (29.3)	135.0 (19.2)	130.3 (20.5)
HOMA-IR	2.5 (1.1)	2.5 (1.0)	2.3 (1.0)

**Table 3:** Glucose AUC, peak, and time to peak values.

Food Matrix	Bread (*n* = 10)				Tortilla (*n* = 10)				Arepa (*n* = 8)		
Soy %	0%	10%	30%	*p*-value	0%	10%	30%	*p*-value	0%	30%	*p*-value
AUC, mg·min/dL × 10^4^ ± SEM	1.43 ± 0.04 ^a^	1.40 ± 0.04 ^a,b^	1.33 ± 0.05 ^b^	**0.03**	1.43 ± 0.07	1.39 ± 0.06	1.34 ± 0.03	0.14	1.46 ± 0.04	1.41 ± 0.04	**0.043**
Peak, mg/dL ± SEM	145.6 ± 5.7 ^a^	138.6 ± 4.6 ^a,b^	130.7 ± 5.0 ^b^	**0.007**	134.2 ± 7.0	131.3 ± 5.4	127.8 ± 4.9	0.30	141.4 ± 4.0	143.2 ± 4.5	0.84
Time to peak, min ± SEM	48.0 ± 6.6	45.0 ± 5.0	36.0 ± 4.0	0.11	57.0 ± 8.3	60.7 ± 11.4	56.8 ± 9.3	0.86	56.3 ± 6.8	48.8 ± 5.5	0.17

Data are presented as mean ± SEM. *p*-value from the repeated measure ANOVA for each individual food matrix; Bolded values represent statistically significant *p*-values (*p* < 0.05). Values with different superscript letters (a, b) differ significantly, whereas values sharing at least one superscript letter are not significantly different (e.g., plasma glucose AUC for control (0%) bread differs significantly from that of 30% soy replacement, but not 10% replacement).

**Table 4:** Insulin AUC, peak, and time to peak values.

Food Matrix	Bread (*n* = 10)				Tortilla (*n* = 10)				Arepa (*n* = 8)		
Soy %	0%	10%	30%	*p*-value	0%	10%	30%	*p*-value	0%	30%	*p*-value
AUC, uU·min/mL × 10^4^ ± SEM	1.02 ± 0.29	0.91 ± 0.15	0.92 ± 0.16	0.36	0.78 ± 0.10	0.77 ± 0.08	0.71 ± 0.09	0.37	0.84 ± 0.14	0.93 ± 0.18	0.48
Peak, uU/mL ± SEM	158.16 ± 33.83	114.11 ± 20.82	127.15 ± 21.76	0.10	95.22 ± 13.78	101.47 ± 12.57	93.23 ± 14.76	0.37	107.96 ± 20.90	129.57 ± 23.66	0.39
Time to peak, min ± SEM	45.0 ± 6.7	48.0 ± 4.9	42.4 ± 6.8	0.71	63.0 ± 5.4	72.0 ± 10.2	66.0 ± 4.0	0.63	63.8 ± 8.9	41.3 ± 5.5	**0.02**

Data are presented as mean ± SEM. *p*-value from the repeated measure ANOVA for each individual food matrix. Bolded values represent statistically significant *p*-values (*p* < 0.05).

## Data Availability

The data presented in this study are available upon request from the corresponding authors for both studies. The data are not publicly available in accordance with the consent provided by participants on the use of confidential data.

## Abbreviations

The following abbreviations are used in this manuscript:

AUC	Area Under the plasma concentration–time curves
